# Ozoralizumab, a trivalent anti-TNFα NANOBODY^®^ compound, is resistant to rheumatoid factor-mediated modulation of FcRn-dependent recycling and immune complex uptake

**DOI:** 10.3389/fimmu.2026.1927219

**Published:** 2026-09-18

**Authors:** Mai Morimoto, Yukihisa Sawa, Chihiro Ishiwatari-Ogata, Naoki Kojima, Yasuyuki Fujii

**Affiliations:** Research Headquarters, Taisho Pharmaceutical Co., Ltd., Saitama, Japan

**Keywords:** anti-TNFα antibody, FcRn recycling, immune complex, phagocytosis, rheumatoid arthritis, rheumatoid factor, tumor necrosis factor, VHH

## Abstract

Rheumatoid factor (RF), an autoantibody targeting IgG, is a key diagnostic marker of autoimmune diseases, especially rheumatoid arthritis (RA). In patients with RA, high RF titers have been reported to be associated with disease severity and may influence the pharmacological behavior and clinical response of certain biologic agents, including tumor necrosis factor (TNF) inhibitors. TNF inhibitors are widely used biologic agents in RA treatment and include two structure types: the fragment crystallizable (Fc)-containing protein (e.g. IgG) and the Fc-free protein. Clinical studies have suggested that in RA patients with high RF titers, sufficient circulating drug concentrations are more likely to be maintained with Fc-free TNF inhibitors than with Fc-containing TNF inhibitors. To clarify the mechanisms underlying this clinical observation, we investigated whether RF differentially alters FcRn-dependent recycling and macrophage uptake of the Fc-containing antibody adalimumab and the Fc-free, human serum albumin (HSA)-binding NANOBODY^®^ compound ozoralizumab (OZR) using an ELISA-based RF-binding assay, an FcRn-mediated recycling assay in FcRn-expressing cells, and a mouse macrophage phagocytosis assay. In a RF binding assay, OZR showed minimal binding to RF. In the FcRn-mediated recycling assay, the recycling efficiency of OZR was not affected by high RF titers. In contrast, adalimumab, an IgG type of TNF inhibitor, exhibited clear interaction with RF and its recycling efficiency decreased in the presence of high RF titers. Furthermore, in a mouse macrophage phagocytosis assay, immune complexes containing the Fc-containing adalimumab, but not the Fc-free OZR, showed increased macrophage uptake in the presence of RF. In summary, our findings demonstrate that the HSA-binding property of OZR enables preservation of HSA-mediated FcRn recycling and avoids RF-enhanced immune-complex phagocytosis under high-RF conditions. These mechanisms may contribute to the maintenance of stable circulating concentrations and therapeutic efficacy of OZR in RA patients with high RF titers.

## 1 Introduction

Rheumatoid arthritis (RA) is a systemic autoimmune disease characterized by chronic and progressive inflammation of the synovial lining of joints leading to synovial hypertrophy, joint swelling, cartilage destruction, and bone erosion (1, 2). Approximately 60%–80% of patients with RA show seropositivity for IgM-type rheumatoid factor (IgM-RF), which is widely used as a diagnostic marker of several autoimmune diseases (3–5). Rheumatoid factor (RF) is an autoantibody that recognizes the fragment crystallizable (Fc) portion of IgG (3–5). While IgM-RF is the predominant isotype, IgA- and IgG-type RFs are also present in patients with RA (3–5). RF can also be detected at low levels in some healthy individuals. Although the presence of autoantibodies in healthy individuals has often been attributed to impaired removal of autoreactive components, growing evidence indicates that such antibodies may play physiological roles in maintaining immune homeostasis (6). Under physiological conditions, low-affinity IgM-RF is transiently induced following stimulation by immune complexes and polyclonal B-cell activators. Such RF has been proposed to promote immune complex clearance by increasing the stability and size of immune complexes and to facilitate antigen presentation to T cells through enhanced uptake of immune complexes by B cells (5). In contrast, the RF derived from patients with RA shows markedly stronger immunological activity (6). Elevated IgM-RF levels have been reported to be associated with higher disease activity and accelerated joint destruction in RA patients (3–5). Accordingly, the European Alliance of Associations for Rheumatology “Treat-to-Target” recommendations classify RF positivity as a poor prognostic factor (7).

RF positivity has been associated with reduced therapeutic responses to biologic agents and other disease-modifying antirheumatic drugs (DMARDs), although treatment outcomes are influenced by multiple clinical and immunological factors, including disease duration, baseline inflammatory burden, genetic factors such as Fcγ receptor polymorphisms, and patient characteristics (8–11). Several studies have reported that tumor necrosis factor (TNF) inhibitors may be less effective in RF-positive patients than in RF-negative patients (8–10), suggesting that RF may be one of several factors associated with treatment response. One potential explanation for this association may be related to the presence of a fragment crystallizable (Fc) portion in some TNF inhibitors (TNFis). In a previous study, significantly lower Simplified Disease Activity Index (SDAI) remission rates were observed among patients with high RF titers receiving Fc-containing TNFis than among those with low RF titers. In contrast, among patients receiving certolizumab pegol (CZP), which is Fc-free, the SDAI remission rates did not differ substantially between patients with high and low RF titers (12). Moreover, among patients with high RF titers, the disease activity was significantly lower in those who were receiving treatment with CZP than in those receiving Fc-containing TNFis (13). While the serum concentrations of CZP remained comparable between patients with high and low RF titers, the serum concentrations of Fc-containing TNFis were reduced in patients with high RF titers (13, 14). Ozoralizumab (OZR) is another Fc-free TNFi (15–17). OZR, a 38-kDa NANOBODY^®^ compound, is a next-generation VHH (variable domain of heavy-chain-only antibody)-based TNFi composed of two anti-human TNF VHH domains and one anti-human serum albumin (HSA) VHH domain. Our previous study has reported that the anti-HSA VHH domain of OZR binds to domain II of HSA. OZR interacts with neonatal Fc Receptor (FcRn) indirectly through its binding to HSA rather than through the canonical IgG Fc-FcRn interaction, thereby prolonging the serum half-life of OZR (17). The Fc-free nature of OZR suggests that, like CZP, OZR may also be less susceptible to RF-mediated interference. In the phase III OHZORA study, significant improvements of disease activity markers, including the Disease Activity Score 28 with C-reactive protein, Disease Activity Score 28 with ESR, Clinical Disease Activity Index, and SDAI, were observed in RA patients with high RF titers (153–2029 IU/mL), and the magnitude of improvement was consistent across RF titers. Moreover, the serum OZR concentrations also did not differ significantly across RF titers (18). These findings suggest that OZR, like CZP, may maintain therapeutic efficacy even in patients with high RF titers. However, despite these clinical observations, the mechanisms underlying the differential effects of RF on Fc-containing and Fc-free TNFis remain unclear. In particular, the direct binding properties between RF and TNFis and the potential consequences for drug pharmacokinetics have not been fully elucidated (6).One proposed mechanism underlying the reduced efficacy of Fc-containing TNFis in patients with high RF titers is that RF may bind to the Fc portion of the TNFis, possibly resulting in the formation of immune complexes that may interfere with antibody recycling or enhance antibody clearance by phagocytic cells (2, 3, 12, 19–21). FcRn plays a critical role in maintaining the serum levels and prolonged half-lives of IgG and HSA (22, 23). After endocytosis, IgG binds to FcRn in acidic endosomes and is protected from lysosomal degradation before being recycled back to the circulation (22, 23). Consistent with this mechanism, treatment with FcRn inhibitors such as efgartigimod results in a marked reduction in circulating IgG concentrations (24). Therefore, factors that alter the interaction between therapeutic antibodies and FcRn may affect antibody pharmacokinetics. In addition, immune complexes formed by RF binding to the Fc portion of therapeutic antibodies may be more readily recognized by phagocytic cells expressing Fc receptors. Larger immune complexes have been reported to undergo more rapid clearance than monomeric antibodies or smaller complexes, potentially reducing circulating drug concentrations (25, 26). Clinical studies have also suggested an association between immune-complex formation and increased clearance of therapeutic antibodies (27, 28). Based on these observations, we hypothesized that Fc-free TNFis, such as OZR and CZP, may be less susceptible to RF-mediated effects related to FcRn-mediated recycling and macrophage-mediated phagocytosis because they do not form RF-Fc immune complexes.

In this study, to experimentally verify this hypothesis, we investigated the binding interactions between RF and representative Fc-containing and Fc-free TNFis and examined the effects of RF on their pharmacokinetics. Adalimumab (ADA) was selected as the representative Fc-containing TNFi because it is one of the most widely used IgG-based TNFis. OZR was selected as the representative Fc-free TNFi because it is a VHH-based TNFi, enabling us to compare the mechanistic differences between IgG-based and VHH-based TNFis. The aim is to clarify, at least partially, the mechanism underlying the maintained therapeutic efficacy of this next-generation NANOBODY^®^ compound even in the presence of high RF titers.

## 2 Materials and Methods

### 2.1 Binding assay for RF derived from human plasma

For the RF binding assay, OZR and ADA were immobilized at a concentration of 1 µg/mL each on immunoplates that were incubated overnight at 4 °C. After washing, the plates were blocked with Blocking/Sample Dilution Buffer (Chondrex, WA, USA) for 1 hour at room temperature. Following additional washing with PBST, RF derived from human plasma (Medix Biochemica, Espoo, Finland, Cat. No. 508-27) was diluted in Blocking/Sample Dilution Buffer to concentrations ranging from 0 to 100 IU/mL, added to the plates, followed by further incubation of the plates for 1.5 hours at room temperature. The plates were then washed with PBST and incubated for 1 hour at room temperature with HRP-conjugated F(ab’)_2_ goat anti-human IgM (heavy chain) secondary antibody (Thermo Fisher, MA, USA). After a final wash with PBST, TMB substrate (Seracare, MA, USA) was added and the absorbance at 450 nm was measured using a microplate reader (Tecan, ZH, CH). All independent experiments within each experimental series were performed using the same RF preparation.

### 2.2 Assessment of antibody binding to FcRn by ELISA

OZR and ADA were immobilized at a concentration of 1 µg/mL each on immunoplates that were incubated overnight at 4 °C. After washing, Blocking/Sample Dilution Buffer (Chondrex, WA, USA) was added and the plates were incubated for 1 hour at room temperature. For the wells coated with OZR, HSA (Sigma-Aldrich, MO, USA, Cat. No. A3782) (10 µM), prepared in Blocking/Sample Dilution Buffer, was added, followed by incubation of the plates for 1 hour at room temperature. RF derived from human plasma (Medix Biochemica, Espoo, Finland, Cat. No. 508-27) was diluted to 0–100 IU/mL in Blocking/Sample Dilution Buffer and added to the wells, and the plates were again incubated for 1 hour at room temperature. FcRn-His tag (produced in-house) prepared in Blocking/Sample Dilution Buffer was then added at a concentration of 0.2 µM for ADA and 30 µM for OZR and the plates were incubated for 1 hour at room temperature. Anti-His-tag mAb-HRP-DirecT (MBL, TOKYO, JP), diluted 1:1000 in Detection Antibody Dilution Buffer, was added, followed by incubation of the plates for 1 hour at room temperature. TMB substrate (Seracare, MA, USA) was then added and the absorbance at 450 nm was measured using a microplate reader (Tecan, ZH, CH).

### 2.3 *In Vitro* FcRn-dependent recycling assay

Human FcRn-overexpressing CHO-K1 cells (Genscript, NJ, USA, Cat. No. M00603) were cultured in Ham’s F-12K (Kaighn’s) Medium and seeded onto 48-well plates one day prior to the assay. OZR and ADA were prepared at 100 nM in HBSS (pH 5.8), with 200 nM of HSA (Sigma-Aldrich, MO, USA, Cat. No. A3782) added additionally for OZR. Purified human RF (Molecular DEPOT, CA, USA, Cat. No. B2010861P) at final concentrations of 0–200 IU/mL was added, and the mixtures were incubated at 37 °C for 1 hour. The antibody–RF mixtures were then added to the cells, followed by incubation of the cells for 1 hour at 37 °C. After removing the supernatant and gently washing the cells, HBSS (pH 7.4) was added, and the cells were incubated for 3 hours at 37 °C. The supernatants were collected, and the antibody concentrations were quantified by ELISA. Human TNFα was immobilized onto plates and incubated overnight at 4 °C. Recycled assay supernatants were incubated in assay plates for 2 hours at room temperature. Anti-OZR antibody, produced by Toray Research Center, Inc., was added for OZR detection and anti-ADA antibody (R&D Systems, MN, USA) for ADA detection, followed by incubation for 2 hours at room temperature. Anti-rabbit IgG HRP-linked antibody (Cell Signaling Technology, MA, USA) was then added and the plates incubated for 1 hour at room temperature. TMB substrate (Seracare, MA, USA) was then applied and the absorbance at 450 nm was measured using a microplate reader (Tecan, ZH, CH).

### 2.4 Mice and preparation of primary mouse macrophage

Male C57BL/6J mice aged 6-8 weeks were purchased from Japan SLC, Inc. (Japan SLC, SHIZUOKA, JP). The animals were housed under controlled temperature (23 °C ± 3 °C), humidity (55% ± 20%), and lighting (12 hours light/dark cycle) conditions. All animal experiments reported here were reviewed and approved by the Institutional Animal Care and Use Committee of Taisho Pharmaceutical Co., Ltd., and were conducted in accordance with the Guidelines for Proper Conduct of Animal Experiments (Science Council of Japan, 2006).

Mice were anesthetized by 2% isoflurane (VIATRIS, PE, USA) plus air inhalation with anesthesia machines (Biomachinery, CHIBA, JP) and euthanized by cervical dislocation under deep anesthetic condition. Then femurs and tibias were collected. Bone marrow cells were flushed from these bones, and red blood cells were removed using RBC Lysis Buffer (pluriSelect, SN, DE). The cells were cultured in DMEM high glucose (Gibco, MA, USA) supplemented with 20 ng/ml mouse M-CSF (Miltenyi Biotec, NW, DE), 10% heat-inactivated fetal bovine serum, and penicillin-streptomycin on 10-cm dish. Culture medium was replaced on days 3 and 6.

### 2.5 *In vitro* mouse macrophage phagocytosis assay

OZR and ADA were labeled with pHrodo Red, Succinimidyl Ester (Invitrogen, MA, USA) in accordance with the manufacturer’s instructions. The labeling efficiencies of OZR and ADA were quantitatively assessed and confirmed to be comparable prior to their use in the experiments. Mouse macrophage cell lines (J774) were cultured in DMEM and seeded onto 48-well plates. Each of the labeled OZR and ADA was mixed with equimolar TNFα, human, recombinant (FUJIFILM Wako Pure Chemical Corporation, Osaka, JP, Cat. No. 207-15261) at a final concentration of 300 nM, followed by the addition of purified human RF (Molecular DEPOT, CA, USA, Cat. No. B2010861P-B) at 0–100 IU/mL or monoclonal RFs, RF61 (Cat. No. RHK25302) and RF-AN (Cat. No. RHK25301) (AntibodySystem Laboratories SAS, Paris, FR) at 0–100nM. The antibody–TNFα–RF mixtures were incubated in DMEM at 37 °C for 1 hour and then added to the cells, followed by incubation of the cells for 5 hours at 37 °C. The cells were harvested, and pHrodo-positive cells were analyzed by flow cytometry (BD Biosciences, NJ, USA). Investigators were not blinded during flow cytometry analysis. Fluorescence compensation was not performed because only a single fluorophore was used. Flow cytometry data were analyzed using FlowJo software (BD Biosciences, NJ, USA). Additionally, fluorescence images were obtained using a fluorescence microscope (Keyence, OSAKA, JP) after staining with Hoechst (Thermo Fisher, MA, USA) and PlasMem (Dojindo Laboratories, KUMAMOTO, JP). The same procedure was also performed for primary mouse macrophages. The endotoxin level of recombinant human TNF-α was confirmed to be below the manufacturer’s specified limit. Albumin from human serum and purified human RF were certified as negative for viral contamination by the manufacturers.

### 2.6 Statistical analysis

Welch’s t-test was used to compare the binding of OZR to FcRn in the presence/absence of RF. The same analysis was performed in the presence and absence of HSA.

One-way ANOVA followed by Dunnett’s test was used to compare the recycled ADA levels across RF concentrations ranging from 0 to 200 IU/mL, with the 0 IU/mL condition set as the control. One-way ANOVA was used to compare the recycled OZR levels across RF concentrations ranging from 0 to 200 IU/mL. Welch’s t-test was used to compare the recycled OZR levels in the presence and absence of HSA.

Welch’s t-test was used to compare the antibody uptake in the presence and absence of TNFα. One-way ANOVA followed by Dunnett’s test was used to compare the phagocytosis of TNFα–ADA immune complexes in the presence of RF concentrations ranging from 0 to 100 IU/mL, with the 0 IU/mL condition set as the control. One-way ANOVA was used to compare the phagocytosis of TNFα–OZR immune complexes in the presence of RF concentrations ranging from 0 to 100 IU/mL. All statistical analyses were performed using GraphPad Prism (GraphPad Software, MA, USA).

## 3 Results

### 3.1 RF derived from human plasma binds to Fc-containing TNFi, but not to OZR, Fc-free TNFi, *in Vitro*

IgM is the predominant immunoglobulin isotype of RF, and IgM-type RF has been reported to bind to the Fc portion of IgG (29). Based on this, we hypothesized that RF would bind to Fc-containing IgG-based TNFis, but not to Fc-free VHH-based TNFi. To verify this hypothesis, we compared the binding reactivities of RF to ADA as a representative IgG-based TNFis, and to OZR as a VHH-based TNFi.

To evaluate RF binding under conditions that mimic the cell surface, reactions were performed at pH7.4, while pH5.8 was used to simulate the endosomal environment in which internalized antibodies encounter FcRn. At pH7.4, RF exhibited strong binding to ADA, with detectable binding even at low concentrations of RF (0.3 IU/mL). In contrast, RF showed minimal binding to OZR even in the presence of high RF concentrations (**Figure 1A**). At pH5.8, RF binding to ADA remained robust and concentration-dependent, whereas RF binding to OZR was again negligible (**Figure 1B**). These results demonstrate that IgM-type RF binds strongly to ADA, an Fc-containing TNFi, but not to OZR, which is an Fc-free TNFi.

**Figure 1:**
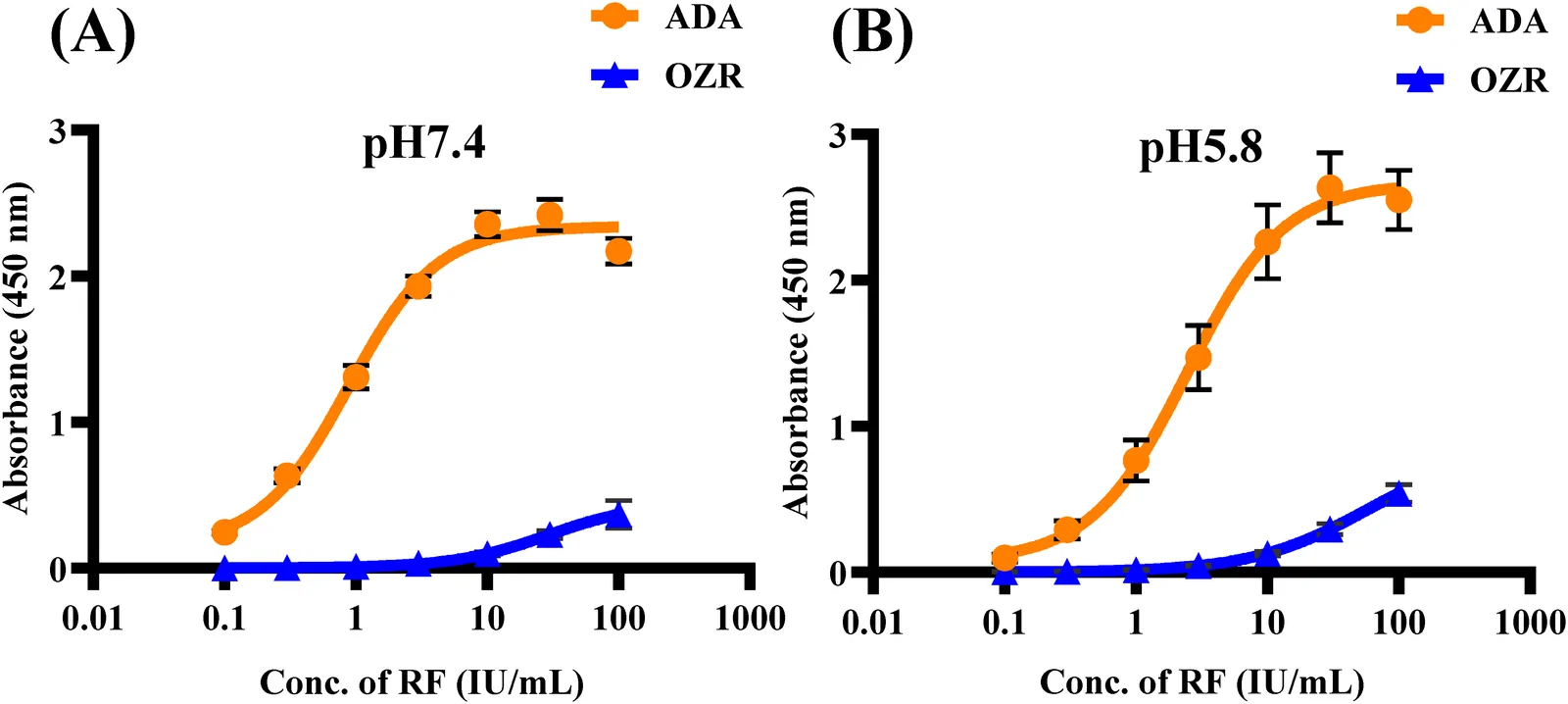
Binding reactivity of ozoralizumab and adalimumab to human rheumatoid factor. The binding reactivity was evaluated by measuring the interaction between the immobilized antibodies on immunoplates and rheumatoid factor. The binding reactivities of ozoralizumab and adalimumab to human rheumatoid factor were assessed at pH7.4 **(A)** and pH5.8 **(B)**. Data are presented as the means of values from three independent biological replicates, and the error bars indicate the standard error of the mean (SEM).

### 3.2 Binding of RF derived from human plasma to Fc-containing TNFi may potentially impact FcRn–TNFi interactions

The serum half-life of both human IgG and HSA is approximately 20 days, and this long half-life of both proteins is maintained through FcRn-mediated recycling in endothelial cells and epithelial cells (22, 23). Because IgG and HSA bind to distinct sites on FcRn, they do not compete with one another and can bind simultaneously to FcRn (23). OZR is a relatively small Fc-free biologic molecule and would therefore be ordinarily rapidly cleared by renal filtration and excretion. However, OZR binds to HSA and exploits the FcRn-mediated recycling pathway of HSA (17), in contrast to IgG antibodies, which rely on their intrinsic Fc portion for FcRn binding. RF may interfere with IgG–FcRn binding by binding to the Fc portion of IgG that is required for the FcRn binding. In contrast, because RF does not bind to HSA, it is unlikely to affect the interaction between HSA and FcRn. Accordingly, we evaluated whether RF might influence the FcRn-mediated recycling of ADA or OZR via HSA.

As expected, binding between FcRn and immobilized ADA was clearly observed (**Figure 2A**). OZR is not designed to bind directly to FcRn, but to interact with it through binding to HSA. Consistent with this notion, OZR binding to FcRn was not observed in the absence of HSA, whereas the presence of HSA enabled OZR–FcRn binding (**Figure 2B**). We next examined whether RF might influence the binding of ADA or OZR to FcRn. When RF derived from human plasma was added to immobilized ADA prior to the addition of FcRn, it inhibited ADA–FcRn binding in a concentration-dependent manner. FcRn binding to ADA was reduced to approximately 55% in the presence of 100 IU/ml of RF as compared with that at the lowest concentration tested (0 IU/mL) (**Figure 2A**). For examining OZR binding to FcRn, HSA was added to immobilized OZR, followed by the addition of RF (100 IU/mL, the concentration exhibiting the strongest inhibition for ADA), and then FcRn. As RF does not bind to either OZR or HSA, the presence of RF had no effect on the HSA-mediated interaction between OZR and FcRn (**Figure 2B**).

**Figure 2:**
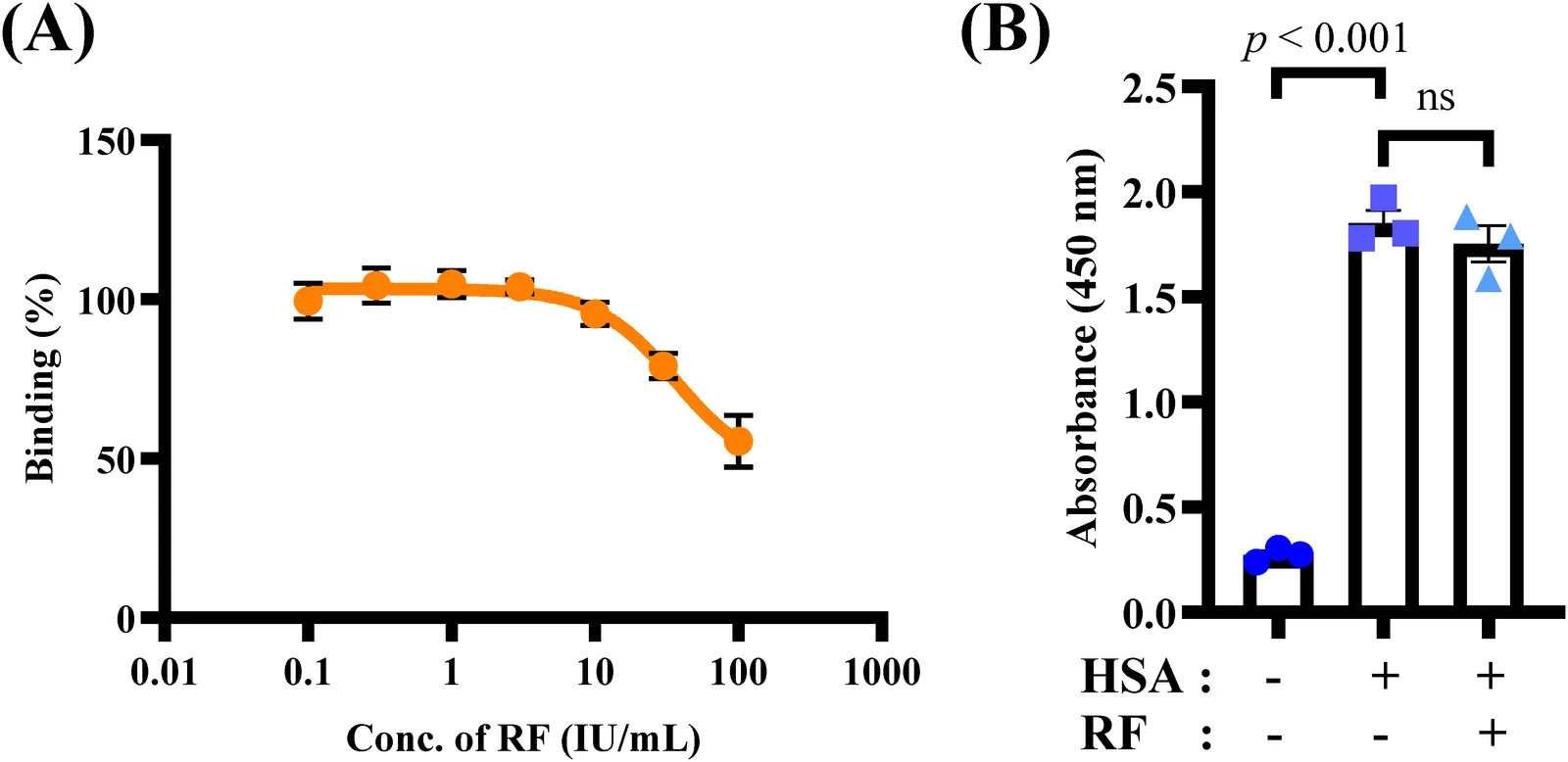
binding reactivity of ozoralizumab and adalimumab to human FcRn in the presence of human rheumatoid factor. The binding reactivity was evaluated by measuring the interaction between the immobilized antibodies on immunoplates and human FcRn protein in the presence of rheumatoid factor. **(A)** binding reactivity of adalimumab to human FcRn in the presence of rheumatoid factor at concentrations ranging from 0 to 100 IU/mL. **(B)** binding reactivity of ozoralizumab to human FcRn in the presence of 100 IU/mL rheumatoid factor with or without human serum albumin. Data represent the means of values obtained from three independent biological replicates, and the error bars indicate the standard error of the mean (SEM). Statistical analyses were performed using Welch’s t-test to compare the binding of OZR in the absence of HSA and RF with that in the presence of HSA, and the binding of OZR in the presence of HSA with that in the presence of both HSA and RF, including for panel **(B)**.

Taken together, these findings indicate that unlike IgG, OZR is recycled via an HSA-mediated mechanism rather than via an Fc-mediated pathway, and that therefore, its recycling efficiency is not affected even in the presence of high RF concentrations.

### 3.3 Recycling of OZR via HSA is minimally affected by the presence of RF

In FcRn-mediated recycling, the binding affinity between the antibodies and FcRn is a critical determinant of the recycling efficiency, and antibodies with low FcRn affinity are known to have shorter serum half-lives (23, 30, 31). OZR is designed to utilize HSA binding for FcRn-mediated recycling and thereby achieve a prolonged serum half-life comparable to that of HSA. However, no published studies have directly demonstrated that OZR undergoes FcRn-mediated recycling through HSA binding.

Therefore, we newly established a cell-based assay system capable of evaluating FcRn-mediated recycling and investigated whether OZR utilizes HSA binding for FcRn-mediated recycling, as well as whether FcRn-mediated recycling of both OZR and ADA are affected by any RF-induced changes in its binding affinity to FcRn.

First, we confirmed that ADA undergoes FcRn-mediated recycling in FcRn-expressing cells (**Figure 3A**). The recycling efficiency of ADA (defined as the amount of recycled ADA divided by the total amount of applied ADA) was approximately 0.47% (data not shown). Previous cell-based IgG recycling assays have reported recycling efficiencies ranging from 0.013% to 0.44% (32–36), although these values depend on the cell type, FcRn expression levels, and incubation time, indicating that this assay is appropriate for evaluating the recycling efficiency. For OZR, while, as expected, no recycling was observed in the absence of HSA, OZR was successfully recycled and detected in the culture supernatant in the presence of HSA (**Figure 3B**). The recycling efficiency of OZR was approximately 0.02% (data not shown). Previous cell-based HSA recycling assays have reported recycling efficiencies ranging from 0.022% to 0.030% (32, 36, 37), indicating that this assay is appropriate for evaluating HSA-mediated recycling efficiency. The recycling efficiency of OZR in the absence of HSA was approximately 0.002% (data not shown), which was significantly lower than that observed in the presence of HSA. These results indicate that OZR undergoes FcRn-mediated recycling through binding to HSA. We then examined whether the presence of RF might affect the FcRn-mediated recycling of the two antibodies. RF inhibited recycling of ADA in a concentration-dependent manner (**Figure 3A****),** whereas no reduction in the recycling efficiency of OZR in the presence of RF was observed (**Figure 3B**).

**Figure 3:**
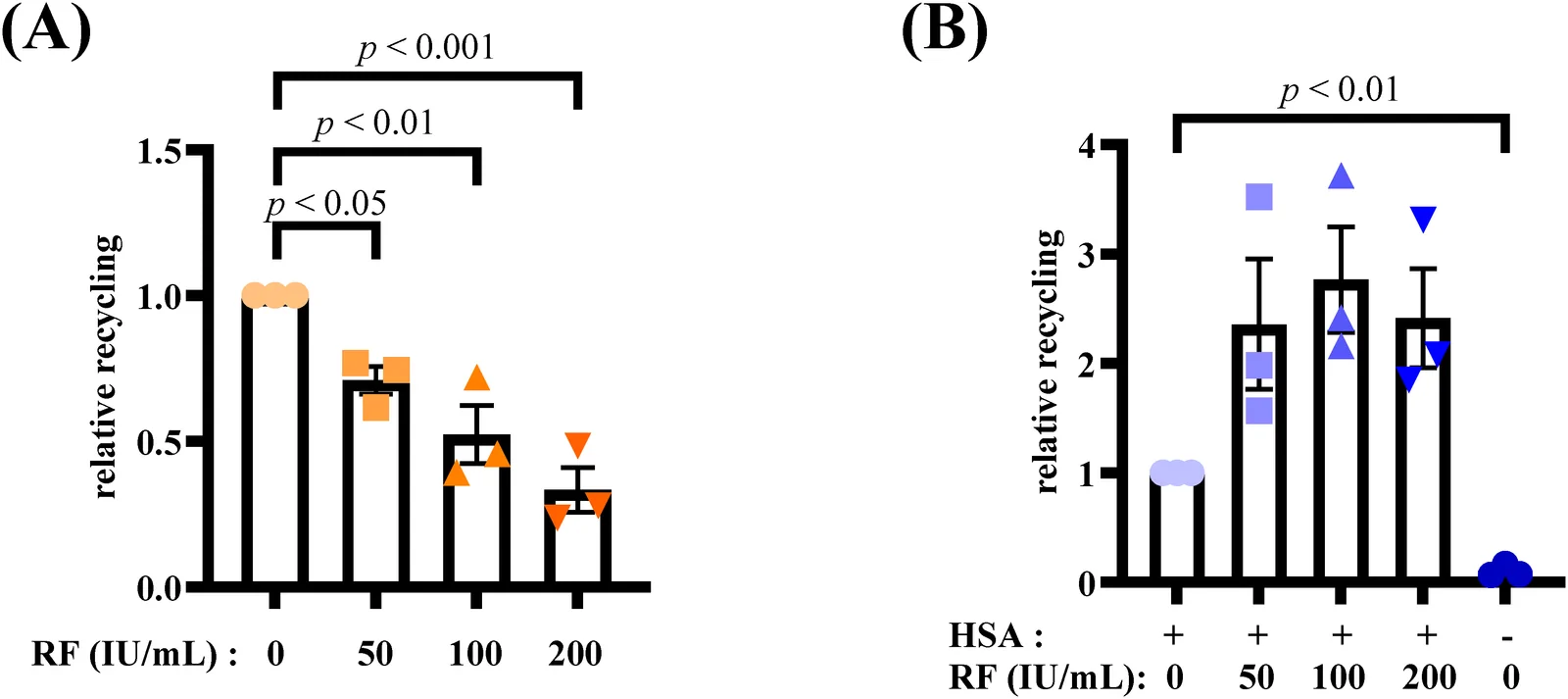
Recycling efficiency of ozoralizumab and adalimumab in the presence of human rheumatoid factor. A mixture of each of the antibodies with rheumatoid factor (0 to 200 IU/mL) was applied to human FcRn-overexpressing CHO-K1 cells, and the concentrations of the recycled antibodies were measured by ELISA. **(A)** Recycling efficiency of adalimumab in the presence of rheumatoid factor concentrations ranging from 0 to 200 IU/mL. **(B)** Recycling efficiency of ozoralizumab in the presence of rheumatoid factor concentrations ranging from 0 to 200 IU/mL. Data represent the means of values from three independent biological replicates, and the error bars indicate the standard error of the mean (SEM). Statistical analysis was performed using one-way ANOVA followed by Dunnett’s test for panel **(A)**, with rheumatoid factor = 0 IU/mL as the control. One-way ANOVA was used for panel **(B)**, across RF concentrations ranging from 0 to 200 IU/mL. Welch’s t-test was used for panel **(B)** to compare recycled OZR levels in the presence and absence of HSA.

Taken together, these findings indicate that RF binds to the Fc portion of ADA, thereby reducing its binding affinity to FcRn and also its recycling efficiency. In contrast, RF does not bind to OZR as OZR lacks an Fc portion and therefore does not interfere with the HSA-mediated interaction between OZR and FcRn, so that OZR recycling is preserved.

### 3.4 RF selectively enhances macrophage uptake of immune complexes containing Fc-containing TNFi

In addition to FcRn-mediated recycling, phagocytic removal of antigen–antibody immune complexes is a key determinant of antibody pharmacokinetics, and large immune complexes are preferentially cleared by macrophages (25, 26). We hypothesized that in the presence of RF, an Fc-containing TNFi with high RF affinity (ADA) might be more susceptible to macrophage-mediated clearance than an Fc-free TNFi with low RF affinity (OZR). To verify this hypothesis, we evaluated the phagocytosis of pHrodo-labeled immune complexes by J774 macrophages.

Based on preliminary experiments, a 5-hour incubation period was selected because antibody uptake was sufficiently detectable at this time point (data not shown). In this study, we used pHrodo, a pH-sensitive fluorescent dye that shows minimal fluorescence at neutral pH and becomes fluorescent in acidic environments. Therefore, the fluorescence signal is expected to be detected mainly after the antibody has been internalized and transported to acidic intracellular compartments, such as endosomes and lysosomes. Minimal phagocytosis was observed for the antibodies themselves, with only approximately 0.1% and 0.3% uptake for ADA and OZR (**Figures 4A, B**), respectively, and OZR uptake was not affected by the presence of HSA (data not shown). However, statistically significantly higher uptake of TNFα–ADA immune complexes (**Figure 4A**), but not of TNFα–OZR immune complexes (**Figure 4B**), was observed. Addition of RF selectively and dose-dependently enhanced phagocytosis of the TNFα–ADA immune complexes (**Figures 4C, E**), with uptake increasing to approximately 17% at the highest RF concentration of 100 IU/mL. In contrast, uptake of the TNFα–OZR immune complexes was not enhanced by RF (**Figures 4D, E**), with uptake remaining at approximately 0.8% even in the presence of 100 IU/mL RF. Immune complex formation between RF and antibodies failed to trigger phagocytic activity in the absence of TNFα (**Supplementary Figure 1**). The RF dependence was corroborated using two other monoclonal RFs (RF61 and RF-AN), which reproduced the selectively enhanced uptake of TNFα–ADA immune complexes, but not of the TNFα–OZR immune complexes (**Supplementary Figure 2**).

**Figure 4:**
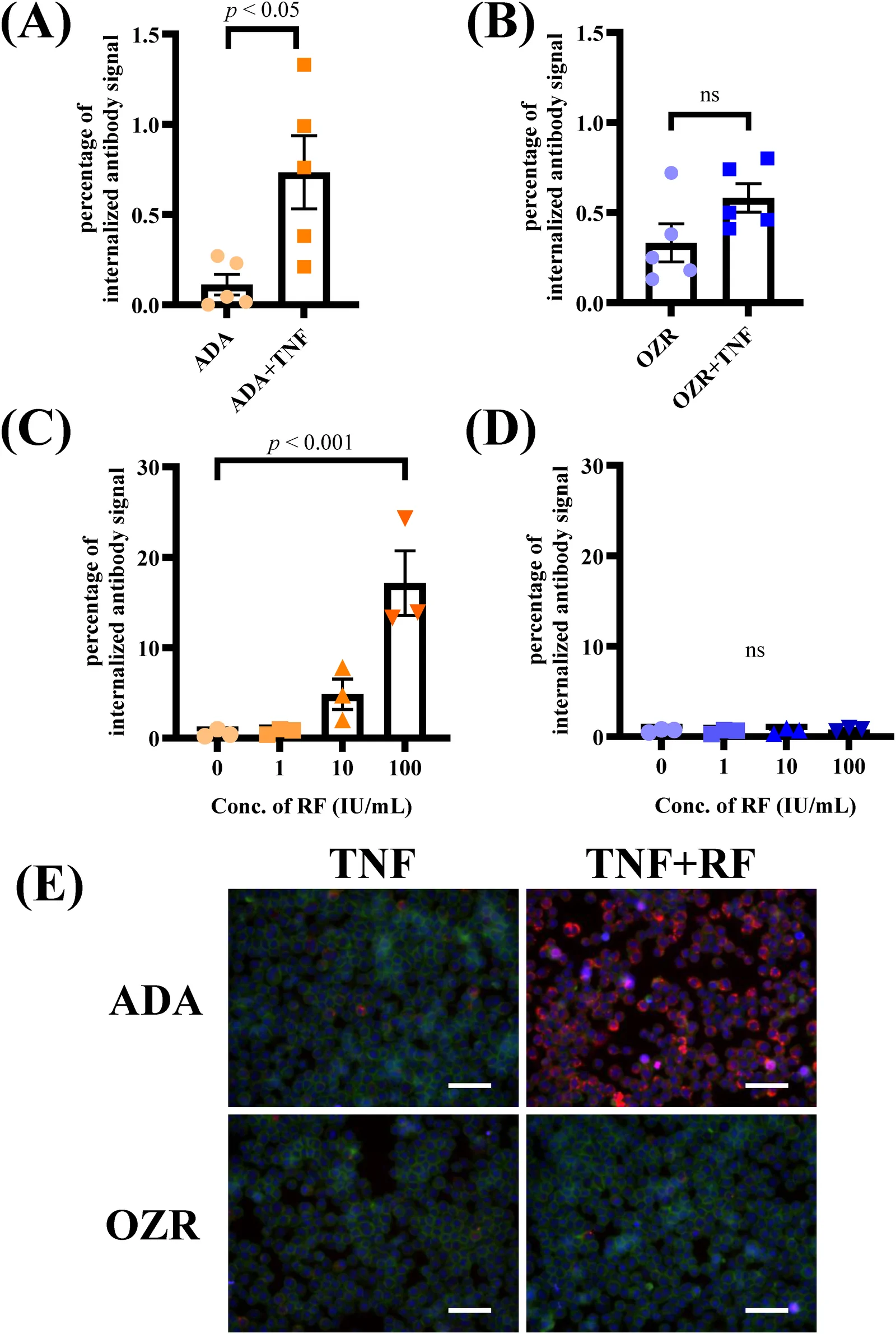
Phagocytic activity of J774 cells for TNFα–ozoralizumab and TNFα–adalimumab immune complexes in the presence of human rheumatoid factor. TNFα–pHrodo-labeled antibody immune complexes were applied to J774 cells and the phagocytic activity was assessed based on the intracellular fluorescence intensity. **(A)** Phagocytic activity of J774 cells for adalimumab and TNFα–adalimumab immune complexes. **(B)** Phagocytic activity of J774 cells for ozoralizumab and TNFα–ozoralizumab immune complexes. **(C)** Phagocytic activity of J774 cells for TNFα–adalimumab immune complexes in the presence of rheumatoid factor at concentrations ranging from 0 to 100 IU/mL. **(D)** Phagocytic activity of J774 cells for TNFα–ozoralizumab immune complexes in the presence of rheumatoid factor at concentrations ranging from 0 to 100 IU/mL. **(E)** Representative fluorescence microscopic images showing phagocytosis of immune complexes by J774 cells in the presence of 100 IU/mL of rheumatoid factor (Green: cell membrane; Red: pHrodo-labeled antibody; Blue: cell nucleus). The scale bar represents 50 μm. Data represent the means of five independent biological replicates for panels **(A)** and **(B)**, and three independent biological replicates for panels **(C, D)**. Error bars indicate the standard error of the mean (SEM). Statistical analyses were performed using Welch’s t-test for panels **(A, B)**. One-way ANOVA followed by Dunnett’s test was used for panel **(C)**, with RF = 0 IU/mL as the control, and one-way ANOVA was used for panel **(D)**.

These findings indicate that RF binds to TNFα–ADA immune complexes and promotes Fc-dependent internalization. However, it has no significant effect on the internalization of TNFα–OZR immune complexes, as it does not bind to the TNFα–OZR immune complex.

### 3.5 RF-mediated enhancement of the uptake of Fc-containing TNFi–antigen immune complexes is conserved in primary mouse macrophages

In J774 cells, we found that the presence of RF enhanced the internalization of Fc-containing TNFi immune complexes. To confirm the physiological relevance of our findings obtained in this cell line, we performed the same experiments using primary cells: we generated primary macrophages from murine bone marrow. First, we assessed the expressions of surface markers on the cells and confirmed that the cells expressed macrophage-associated markers, including CD11b and F4/80 (data not shown). In the primary macrophages, RF, as in the J774 cells, enhanced the phagocytosis of TNFα–ADA immune complexes. Specifically, the phagocytosis of TNFα–ADA immune complexes were approximately 0.3% in the absence of RF and increased markedly to approximately 30% in the presence of 100 IU/mL RF. In contrast, this RF-dependent enhancement in uptake was not observed for TNFα–OZR immune complexes (**Figures 5A–C**). The uptake of TNFα–OZR immune complexes was approximately 0.3% in the absence of RF and remained nearly unchanged at approximately 0.4% even in the presence of 100 IU/mL RF. These results further confirmed that RF exerts no effect on macrophage-mediated phagocytosis of TNFα–OZR immune complexes by primary macrophages.

**Figure 5:**
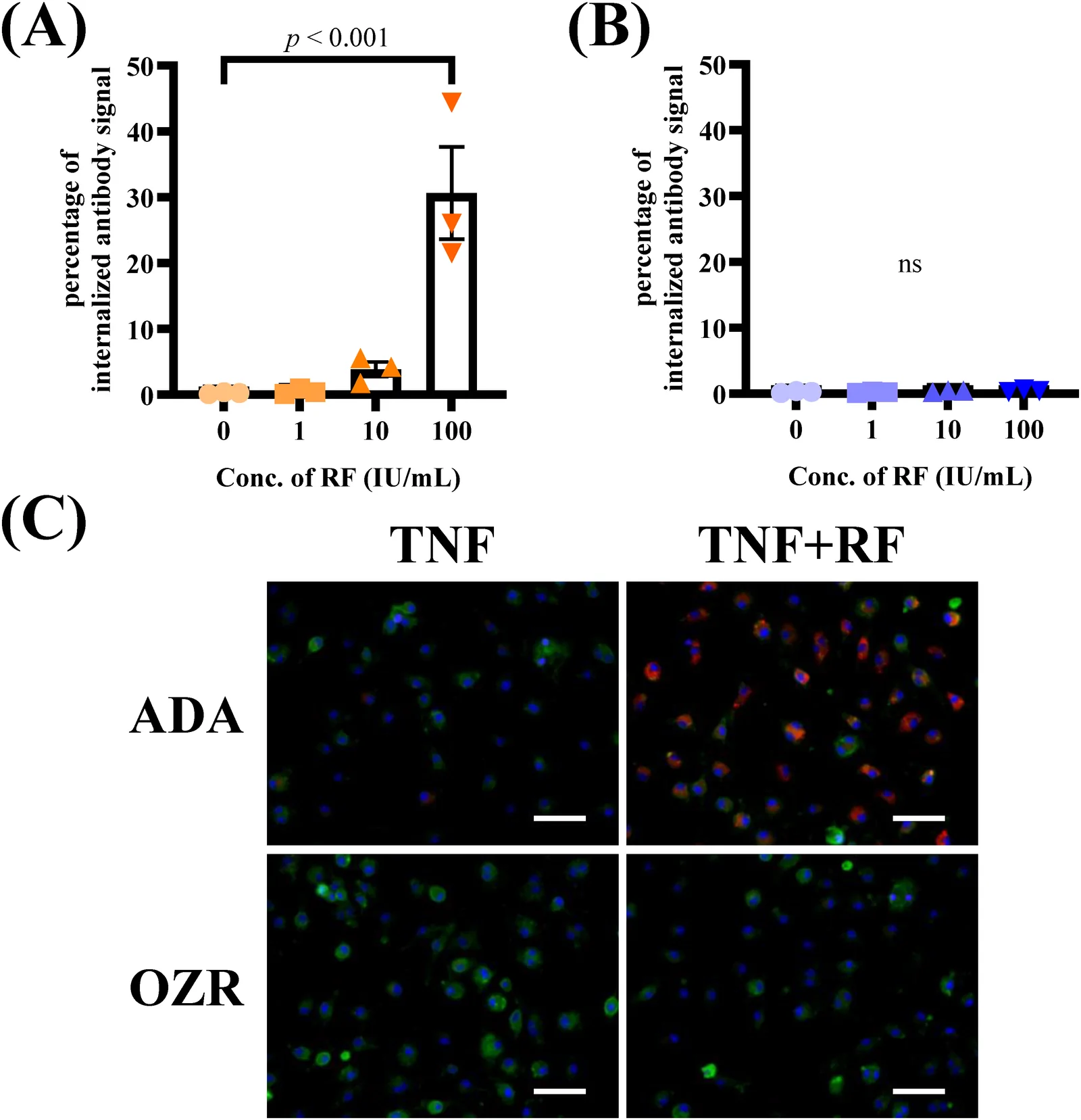
Phagocytic activity of primary macrophages for TNFα–ozoralizumab and TNFα–adalimumab immune complexes in the presence of human rheumatoid factor. TNFα–pHrodo-labeled antibody immune complexes were applied to primary macrophages, and the phagocytic activity was evaluated based on intracellular fluorescence intensity. **(A)** Phagocytic activity of primary macrophages for TNFα–adalimumab immune complexes in the presence of rheumatoid factor at concentrations ranging from 0 to 100 IU/mL. **(B)** Phagocytic activity of primary macrophages for TNFα–ozoralizumab immune complexes in the presence of rheumatoid factor at concentrations in the range of 0 to 100 IU/mL. **(C)** Representative microscopic images showing phagocytosis of immune complexes by primary macrophages in the presence of 100 IU/mL of rheumatoid factor (Green: cell membrane; Red: pHrodo-labeled antibody; Blue: cell nucleus). The scale bar represents 50 μm. Data represent the means of three independent biological replicates, and the error bars indicate the standard error of the mean (SEM). Statistical analyses were performed using one-way ANOVA followed by Dunnett’s test for panel **(A)**, with rheumatoid factor (RF) = 0 IU/mL as the control, and one-way ANOVA was used for panel **(B)**.

## 4 Discussion

RF is a polyclonal autoantibody that recognizes the Fc portion of IgG. Although treatment resistance in RA is influenced by multiple factors, high RF titers have been reported to be one of the factors associated with a poor prognosis, accelerated disease progression, increased joint and bone destruction, and reduced therapeutic responses to biological DMARDs (8–11). Tanaka et al. reported that patients with high RF titers (>204 IU/mL) exhibited lower response rates to ADA as compared to patients with the lowest RF titers. In contrast, among patients treated with CZP, an Fc-free PEGylated Fab fragment, the clinical outcomes were comparable regardless of the RF titers (38). Similarly, in RA patients receiving a TNFi (infliximab (IFX), ADA, or CZP), the serum drug concentrations measured at 6 months after treatment initiation were decreased in patients with high RF titers (>380 IU/mL) as compared with RF-negative patients (RF < 20 IU/mL) in the IFX and ADA, but not the CZP, groups; the serum CZP concentrations were not significantly influenced by the baseline RF titers (14). In the *post hoc* analysis of the OHZORA phase II/III study, patients stratified by quartiles of the baseline RF showed significant reductions in disease activity across all quartiles following treatment with ozoralizumab (OZR) 30 mg. Unlike the case for Fc-containing IgG-type TNFis, whose serum concentrations decrease in patients with high RF titers (13, 14), the serum concentrations of OZR were maintained for up to 52 weeks even in patients with high RF titers. Taken together, these findings suggest that OZR may be effective in patients with RA across different RF titers (18).

Several mechanisms have been proposed to explain the RF-mediated reduction of serum Fc-containing TNFi concentrations. One potential mechanism is that IgM-RF may bind to the Fc portion of IgG-based TNFis, and thereby (i) interferes with Fc portion-FcRn interactions that are essential for IgG recycling and (ii) promotes the formation of large immune complexes that are rapidly cleared by endocytosis and possibly facilitate the generation of anti-drug antibodies. These processes may collectively contribute to reducing circulating drug levels and attenuating therapeutic efficacy (14, 19, 38).

OZR is a trivalent humanized small-molecule antibody consisting of two anti-TNFα NANOBODY^®^ domains and one anti-HSA NANOBODY^®^ domain (17). Similar to CZP, OZR is an Fc-free TNFi. The half-life extension of OZR relies on HSA-mediated engagement with FcRn, rather than direct binding of OZR to FcRn. This mechanism is distinct from the canonical Fc–FcRn interaction that prolongs the serum half-life of IgG. The anti-HSA NANOBODY^®^ domain binds to domain II of HSA, whereas HSA interacts with FcRn primarily through domains I and III. Because the OZR-binding site and FcRn-binding sites on HSA are spatially distinct, HSA is thought to retain its ability to bind FcRn even when associated with OZR (17). In contrast, IgG binds FcRn via the CH2 and CH3 interface of Fc portion. The FcRn-binding sites for IgG and HSA do not overlap, allowing simultaneous engagement with FcRn (23, 30). High affinity for FcRn is critical for efficient recycling, and loss of FcRn binding markedly shortens the serum half-life (23, 30, 31, 37, 39). Since RF recognizes the CH2–CH3 portion of IgG (40), which partially overlaps with the FcRn-binding site (23), it has the potential to interfere with FcRn-mediated recycling of IgG-based therapeutics.

HSA-based half-life extension strategies have been extensively investigated (37, 41, 42). Several approaches, such as fatty-acid modification to promote HSA binding (e.g., liraglutide) or direct HSA fusion, also exploit HSA binding to prolong the half-life (43). For these agents, the increased molecular size upon HSA binding is known to reduce renal clearance; however, FcRn-mediated recycling has not yet been experimentally demonstrated. Although HSA-fusion proteins have been reported to utilize FcRn-mediated recycling (37, 41), no study has shown that a therapeutic molecule equipped with an HSA-binding domain can bind endogenous HSA and subsequently undergo FcRn-mediated recycling (44). Our findings provide the first direct evidence that OZR leverages endogenous HSA to undergo FcRn-mediated recycling, representing the first experimental demonstration of this mechanism in HSA-binding therapeutics. Moreover, OZR showed minimal binding to RF. Consistent with this finding, RF had no appreciable effect on either the interaction between OZR and FcRn via HSA or HSA-mediated FcRn recycling of OZR. Although minimal RF binding to OZR was observed at the highest RF concentration tested (100 IU/mL), suggesting that OZR–RF immune complexes could potentially form, RF did not appreciably affect FcRn binding to HSA-associated OZR or HSA-mediated FcRn recycling of OZR under this condition. Thus, although these experiments do not definitively exclude all possible effects of RF-containing complexes, any indirect influence of such complexes on the HSA–FcRn interaction and FcRn-mediated recycling of OZR is likely to be limited under the conditions tested.

Because the RF-binding epitope is located within the CH2-CH3 portion of the Fc portion, it was structurally anticipated that OZR, which lacks the Fc portion, would not be susceptible to RF-mediated impairment of FcRn-mediated recycling. However, to our knowledge, this is the first study to experimentally demonstrate that an Fc-free, HSA-binding therapeutic can avoid RF-mediated impairment of HSA-mediated FcRn recycling. Because RF has been reported to show little or no binding to CZP (45), the minimal binding of RF to OZR is also likely attributable to its Fc-free nature rather than to a mechanism specific to OZR. In contrast, the ability of OZR to maintain recycling in the presence of RF may be attributable not only to its Fc-free nature but also to its HSA-binding domain, which enables OZR to interact with FcRn through endogenous HSA. Further studies comparing OZR with other Fc-free biologics employing alternative half-life extension technologies will be required to determine the relative contribution of these factors more precisely.

In contrast, RF exhibited dose-dependent binding to ADA. This interaction reduced the binding affinity between ADA and FcRn, particularly in the presence of relatively high RF concentrations. Furthermore, ADA-mediated recycling was also partially inhibited in the presence of high RF titers. These findings suggest that RF-induced reductions in FcRn affinity lead to decreased recycling efficiency. Previous studies have suggested that RF binding may be enhanced when IgG undergoes conformational changes upon ligand engagement, thereby exposing Fc epitopes recognizable by RF (3, 40). In contrast, our findings demonstrate that RF inhibited ADA recycling even in the absence of TNFα, indicating the presence of a distinct mechanism from the mechanisms reported previously. The RF preparation used in this study consisted predominantly of IgM-RF, with IgG-RF accounting for approximately 10% (data not shown). IgG-RF, which undergoes affinity maturation and therefore exhibits higher affinity, has been associated with more severe and refractory RA, including vasculitis (46), whereas IgM-RF is the most prevalent subtype and is commonly used as a disease marker (5). While low-affinity RF likely contributes to protective immune responses, high-affinity and disease-associated RF may stabilize IgG and promote the formation of pathogenic immune complexes (6). Thus, high-affinity RF likely contributes to impaired antibody recycling, particularly when its binding epitopes overlap with the IgG-FcRn interaction interface. Consistent with this speculation, a monoclonal IgG antibody specific for the Fc portion also inhibited FcRn-mediated recycling (data not shown). However, it still remains unclear which RF isotype might contribute predominantly to the inhibition of FcRn-mediated recycling.

The second proposed mechanism involves phagocytosis, which plays a key role in regulating antibody persistence (26). Larger immune complexes are phagocytosed more efficiently by macrophages (47, 48). Previous reports have shown that anti-IFX antibodies induce the formation of immune complexes of varying sizes, with large complexes showing accelerated clearance (26, 49). Anti-immunogenicity strongly influences the efficacy of biologics, and immune complex formation between IFX and anti-IFX antibodies reduces the circulating drug concentrations (27). These observations suggest that RF binding to immune complexes formed by Fc-containing TNFis may enhance their size, thereby promoting rapid clearance via endocytosis, enhancing immunogenicity, and ultimately accelerating overall drug clearance; further investigations are warranted.

In the present study, macrophage phagocytosis of TNFα–ADA immune complexes was enhanced in the presence of high RF concentrations. These findings were consistent across both cell lines and primary cultured cells. A similar enhancement was observed with monoclonal IgM-RFs (RF61 and RF-AN), indicating that IgM-RF can bind ADA and promote macrophage-mediated clearance. These results suggest that large immune complexes formed in the presence of IgM-RFs are more efficiently phagocytosed by macrophages. Although this process may contribute to accelerated clearance, reduced circulating drug levels, and potentially enhanced immunogenicity, these downstream effects were not directly examined in the present study and therefore represent a limitation. It is noteworthy that phagocytosis of immune complexes formed by RF and ADA was not observed in the absence of TNFα, indicating that TNFα is required for the formation of complexes large enough to be phagocytized. In other words, RF binding to ADA alone is insufficient to generate complexes of adequate size for macrophage uptake. According to a previous study, ADA and TNFα interact via multivalent binding (avidity), forming large oligomeric immune complexes rather than simple 1:1 complexes (15). RF can bind to immune complexes containing multiple ADA molecules, further increasing their size and promoting phagocytosis.

In contrast, RF failed to enhance phagocytosis of OZR, regardless of the presence or absence of TNFα. A previous study showed that, even in the presence of TNFα, OZR does not form large immune complexes via multivalent binding due to its structural properties (15). If large immune complexes had formed, enhanced macrophage uptake of OZR would have been expected. Therefore, our findings do not support the extensive formation of large OZR–RF immune complexes and suggest that neither TNFα nor RF is likely to affect macrophage-mediated uptake of OZR. Consistent with these findings, CZP, another Fc-free TNFi, has also been reported not to form large immune complexes with IgM-RF, with minimal uptake by macrophages (45). These observations suggest that the observed effects are more likely attributable to the Fc-free nature of OZR than to structural features unique to OZR. Because OZR lacks an Fc portion, its uptake by macrophages cannot be explained by the conventional Fcγ receptor (FcγR)-mediated pathway responsible for phagocytosis of Fc-containing immune complexes. Although the mechanisms underlying cellular uptake of Fc-free VHH-based molecules remain incompletely understood, FcγR-independent pathways such as fluid-phase pinocytosis, nonspecific endocytosis, or HSA-associated uptake may contribute. However, macrophage uptake of OZR was extremely limited, with only approximately 0.3% uptake for OZR, whereas uptake of TNFα–ADA immune complexes in the presence of high concentrations of RF increased to approximately 17%. These findings indicate that OZR is internalized only minimally compared with Fc-containing immune complexes and support the conclusion that OZR does not undergo efficient FcγR-mediated uptake. Furthermore, although OZR contains an HSA-binding domain, HSA-mediated uptake is unlikely to contribute substantially to OZR internalization. Taken together, these results suggest that the small amount of intracellular OZR detected in macrophages is most likely attributable to basal Fc-independent processes, such as fluid-phase pinocytosis or nonspecific endocytosis.

In summary, our results suggest that OZR is less susceptible to RF-mediated suppression of FcRn-dependent recycling and RF-mediated enhancement of phagocytosis by macrophages under the experimental condition examined in this study, thereby potentially contributing to stable pharmacokinetics in patients with high RF titers. These findings provide a possible mechanistic explanation for the previously reported maintenance of OZR plasma concentrations and clinical responses across different RF titer groups. Further clinical studies are needed to determine the extent to which these mechanisms contribute to treatment outcomes in patients with RA.

## Data Availability

The raw data supporting the conclusions of this article will be made available by the authors, without undue reservation.
