# Supplementary material for: Ozoralizumab, a trivalent anti-TNFα NANOBODY® compound, is resistant to rheumatoid factor-mediated modulation of FcRn-dependent recycling and immune complex uptake
Source: Front Immunol. 2026 Sep 18;17:1927219. doi: 10.3389/fimmu.2026.1927219 (PMC13631012; doi:10.3389/fimmu.2026.1927219)
Supplement: Supplementary file 1 [file DataSheet1.pdf]

Supplementary Material

(A)

(B)

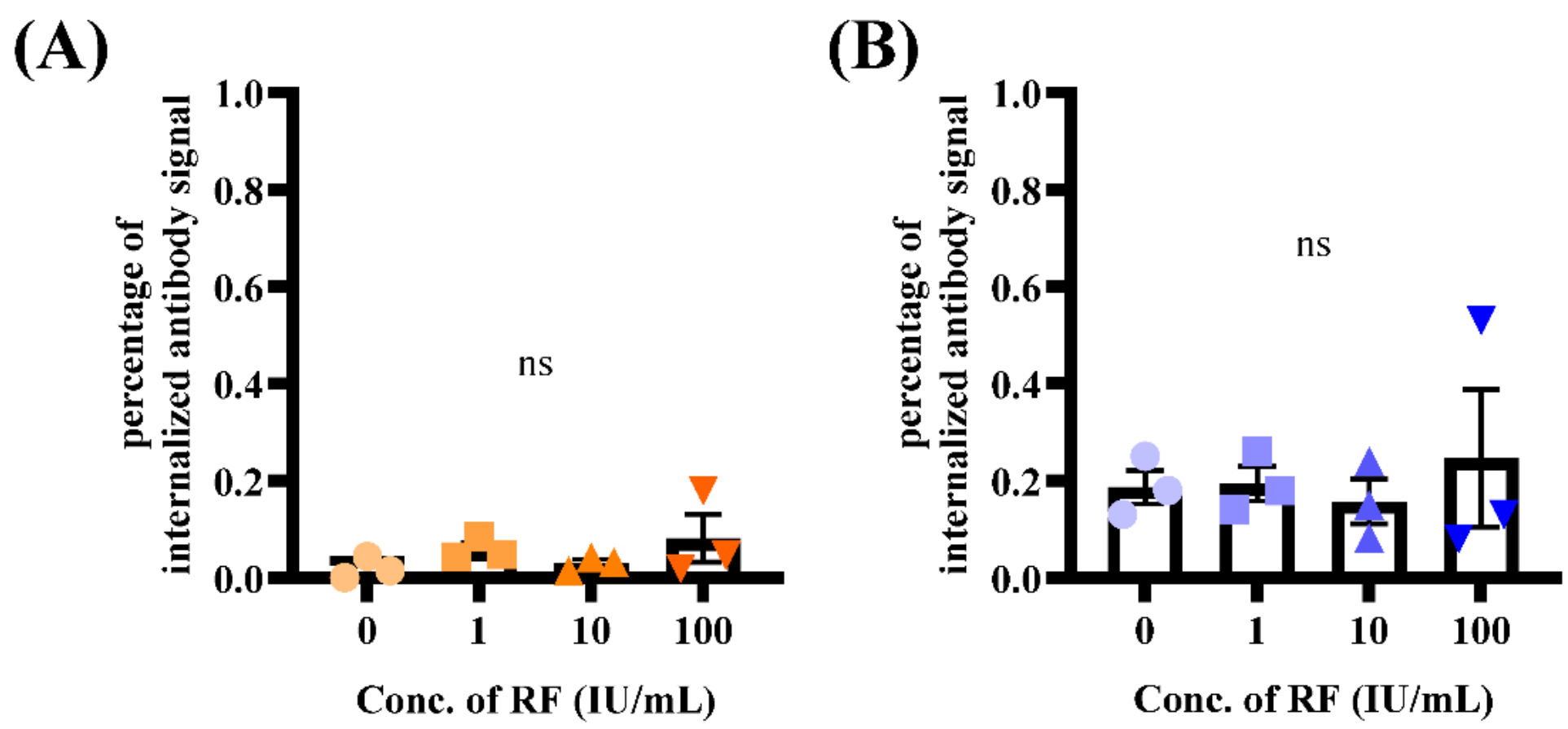

Supplementary Figure 1. Phagocytic activity of J774 cells for ozoralizumab and adalimumab in the presence of human rheumatoid factor.

(A) Phagocytic activity of J774 cells for adalimumab in the presence of rheumatoid factor at concentrations ranging from 0 to 100 IU/mL. (B) Phagocytic activity of J774 cells for ozoralizumab in the presence of rheumatoid factor at concentrations ranging from 0 to 100 IU/mL. Data represent the means of three independent experiments, and the error bars indicate the standard error of the mean (SEM). Statistical analysis was performed using one-way ANOVA for panels (A) and (B).

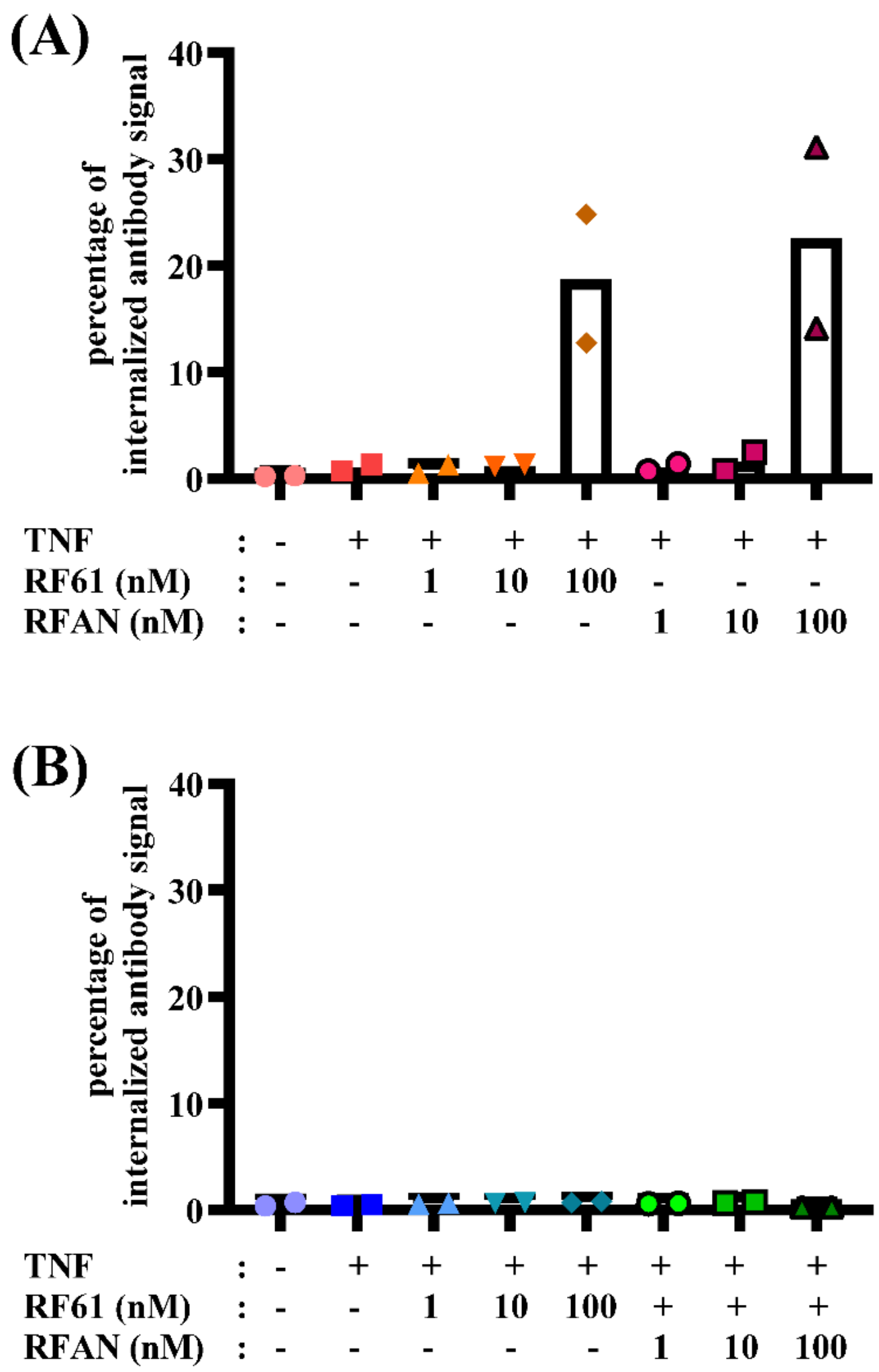

Supplementary Figure 2. Phagocytic activity of J774 cells for TNFα–ozoralizumab and TNFα–adalimumab immune complexes in the presence of monoclonal rheumatoid factors (RF61 and RF-AN).

(A) Phagocytic activity of J774 cells for TNFα–adalimumab immune complexes in the presence of monoclonal rheumatoid factors at concentrations ranging from 0 to 100 nM. (B) Phagocytic activity of J774 cells for TNFα–ozoralizumab immune complexes in the presence of monoclonal rheumatoid factors at concentrations ranging from 0 to 100 nM. Data represent the means of two independent experiments for panels (A) and (B).
